# Altered amygdala and hippocampus effective connectivity in mild cognitive impairment patients with depression: a resting-state functional MR imaging study with granger causality analysis

**DOI:** 10.18632/oncotarget.15335

**Published:** 2017-02-15

**Authors:** Li Juan Zheng, Gui Fen Yang, Xin Yuan Zhang, Yun Fei Wang, Ya Liu, Gang Zheng, Guang Ming Lu, Long Jiang Zhang, Ying Han

**Affiliations:** ^1^ Department of Medical Imaging, Medical Imaging Center, Jinling Hospital, Medical School of Nanjing University, Nanjing 210002, China; ^2^ College of Civil Aviation, Nanjing University of Aeronautics and Astronautics, Nanjing, Jiangsu, 210016, China; ^3^ Center of Alzheimer’s Disease, Beijing Institute for Brain Disorders, Beijing, 100053, China; ^4^ Department of Neurology, Xuanwu Hospital, Capital Medical University, Beijing, 100053, China

**Keywords:** major depression disorder, amnestic cognitive impairment, granger causality analysis, amygdala, hippocampus

## Abstract

Neuroimaging studies have demonstrated that the major depression disorder would increase the risk of dementia in the older with amnestic cognitive impairment. We used granger causality analysis algorithm to explore the amygdala- and hippocampus-based directional connectivity patterns in 12 patients with major depression disorder and amnestic cognitive impairment (mean age: 69.5 ± 10.3 years), 13 amnestic cognitive impairment patients (mean age: 72.7 ± 8.5 years) and 14 healthy controls (mean age: 64.7 ± 7.0 years). Compared with amnestic cognitive impairment patients and control groups respectively, the patients with both major depression disorder and amnestic cognitive impairment displayed increased effective connectivity from the right amygdala to the right lingual and calcarine gyrus, as well as to the bilateral supplementary motor areas. Meanwhile, the patients with both major depression disorder and amnestic cognitive impairment had enhanced effective connectivity from the left superior parietal gyrus, superior and middle occipital gyrus to the left hippocampus, the z values of which was also correlated with the scores of mini-mental state examination and auditory verbal learning test-immediate recall. Our findings indicated that the directional effective connectivity of right amygdala - occipital-parietal lobe – left hippocampus might be the pathway by which major depression disorder inhibited the brain activity in patients with amnestic cognitive impairment.

## INTRODUCTION

Amnestic mild cognitive impairment (aMCI) is defined as the pre-dementia stage with a prominent memory impairment [1, 2]. It has shown that the major depression disorder (MDD) and aMCI have high risk for progression to Alzheimer’s disease (AD), however, the neurobiological mechanisms of this link remain unclear [3, 4]. Commonly, the patients with MCI and depression have the common cognitive dysfunction profiles [5, 6] and the similar functional alteration pattern, particularly in the medial temporal lobe [7]. These findings indicated the similar neuropathological pathway between MCI and depression [4, 6, 7]. Nevertheless, the patients with MDD and aMCI have been reported with greater neurocognitive dysfunctions, grey matter volume loss, as well as disrupted functional integration and segregation compared to the non-depressed MCI [8–11]. These observations underlie the different pathophysiological mechanisms between aMCI with and without MDD, which might be a potentially useful biomarker to identify MCI patients who are the most likely to progress to AD [12].

Neuroimaging studies showed that the alteration of the hippocampus was a common pathway in patients with aMCI and MDD [4, 7]. Hippocampus is central to the episodic memory encoding and retrieve [13]. It has been demonstrated the abnormal activation, volume and metabolism of hippocampus in patients with aMCI [4, 14–16]. Meanwhile, the deregulation of emotional processing is considered the core feature of MDD [17]. The hippocampus is involved in the dorsal-cognitive system of the mood regulation circuitry [4, 18], while the amygdala is the crucial hub of the emotional processing neural circuit [17]. Patients with MDD have shown the disrupted functional and structural connectomes of the amygdala with increased metabolism [17, 19]. These findings implicated that both hippocampus and amygdala were critical in adapting to the pathology of MDD-aMCI.

Resting-state functional magnetic resonance imaging (rs-fMRI) has been widely used to depict the human neural functional architecture and the pathophysiology of many brain diseases [7, 11, 20, 21]. The globally diminished hippocampus-based connectivity and the increased centrality of the amygdala have been reported in MDD [20] and aMCI [23], respectively. Xie et al detected the significant interactions of MDD and aMCI on the hippocampus networks in elderly patients [6], emphasizing its critical role in mood regulation and higher-order cognitive functions. However, how hippocampus and amygdala directly disturbed each other in patients with both aMCI and MDD remain unclear. This study thus investigated the effective connectivity patterns from and to the amygdala and hippocampus by using granger causality analysis, a powerful method widely used for identifying directed functional interactions from time-series data, and revealing the causal effects among brain regions [24]. To the best of our knowledge, this is the first study to examine the time-directed dynamic relations in aMCI-MDD patients using fMRI with granger causality analysis algorithm, which might strengthen our understanding of the neural basis of MDD on aMCI.

## RESULTS

### Demographical and neuropsychological results

The demographics and neuropsychological characteristics are shown in Table 1. There were no differences for age, sex, educational level and all neuropsychological tests among the three groups (all P > 0.05). As expected, there was significant difference in MMSE, MoCA, AVLTs, CDR, and CDT among the three groups (all P < 0.05).

**Table 1:** Demographic, clinical and neuropsychological data of the subjects in this study

	MDD-aMCI(n=12)	aMCI(n=13)	Controls(n=14)	PValue
Age (y)	69.5 ± 10.3	72.7 ± 8.5	64.7 ± 7.0	0.319^c^
Gender (M/F)	5/7	5/8	4/10	0.764^b^
Education (y)	11 (13-9.3)	9 (10.5 - 3)	10 (12-6)	0.309^a^
MMSE/30	27.2 ± 1.7	21.9 ± 4.9	27 (29.3-27)	0.041^a^*
MoCA/30	22.5 ± 3.3	17.9 ± 5.0	25.5 ± 5.7	0.014^c^*
AVLT-immediate recall (n)	4.2 ± 2.1	3.6 ± 1.4	7.3 ± 1.7	0.024^c^*
AVLT-delayed recall (n)	5 (7.0-4.3)	2.9 ±3.3	9.9 ± 2.6	0.004^a^**
AVLT-recognition (n)	7 (12.5-4.3)	7.5 ±3.7	11.3 ± 2.4	0.089^a^
CDR (score)	0.5	0.5	0	-
CDT (score)	3 (3.0-2.0)	2 (2.5-1.5)	3 (3.0-2.75) (7.0-4.3)	0.031^a^*

Mean ± standard deviation; Median and inter-quartile range [M (QU-QL)].

^a^ k-independent samples nonparametric tests; ^b^ chi square test; ^c^ analysis of variance;

* P < 0.05, ** P < 0.01;

Abbreviations: MDD-aMCI, Major Depression Disorder and Amnestic Mild Cognitive Impairment; aMCI, Amnestic Mild Cognitive Impairment; n, number; MMSE, Mini-Mental State Examination; MoCA, Montreal Cognitive Assessment; AVLT, Auditory Verbal Learning Test (AVLT); CDR, Clinical Dementia Rating; CDT, Clock Drawing Test.

### Effective connectivity from the left and right amygdala

**Figure 1:**
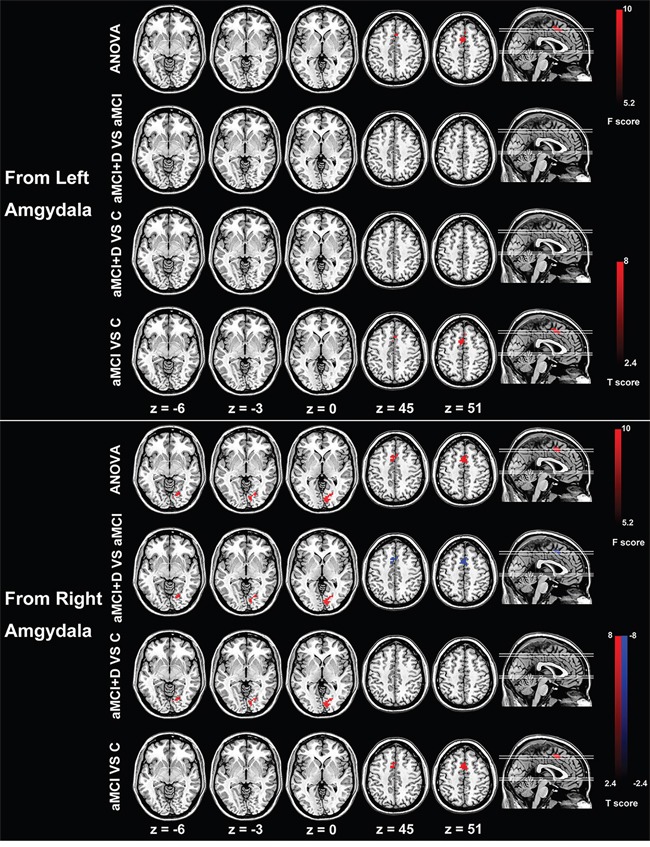
Group differences of effective connectivity based on the seed of right and left amygdala ANOVA analysis shows significant differences of right amygdala-based effective connectivity in right lingual and calcarine gyrus and bilateral supplementary motor areas among the three groups. Compared with the healthy controls, the MDD-aMCI (major depression disorder - amnestic mild cognitive impairment) patients have increased effective connectivity from the right amygdala to the right lingual/calcarine gyrus, the patients with aMCI display enhanced effective connectivity from the right amygdala to the bilateral supplementary motor area compared with the aMCI group. The patients with MDD-aMCI have increased effective connectivity from the right amygdala to the right lingual/calcarine gyrus and decreased effective connectivity to the bilateral supplementary motor areas compared with the aMCI group. Based on the seed of the left amygdala, there is significant difference in effective connectivity from right amygdala to the bilateral supplementary motor areas among the three groups. Compared with healthy controls, the patients with aMCI display increased effective connectivity from the right amygdala to the bilateral supplementary motor areas. There is no difference between the MDD-aMCI and healthy controls and between MDD-aMCI and aMCI groups. The t value and F value color-coded scale are reported at the right side of the images. Abbreviations: ANOVA, analysis of variance; D, major depressive disorder; aMCI, amnestic mild cognitive impairment; C, healthy control.

Based on the seeds of the bilateral amygdala, the effective pattern of the left amygdala to the rest regions was different from that of the right amygdala. From the right amygdala, the right lingual gyrus, the right calcarine gyrus and bilateral supplementary motor areas were detected with significant difference among the three groups. Compared with the healthy control group, the MDD-aMCI group exhibited significantly increased effective connectivity from the right amygdala to the right lingual and calcarine gyrus, and aMCI group displayed increased effective connectivity from the right amygdala to bilateral supplementary motor areas. Besides, the MDD-aMCI group had significantly increased effective connectivity to right lingual and calcarine gyrus, and decreased effective connectivity to bilateral supplementary motor areas compared with the aMCI group. However, based on the seed region of the left amygdala, only the effective connectivity from the left amygdala to bilateral supplementary motor areas was detected with significant difference among the three groups. Compared with the healthy group, aMCI group displayed increased effective connectivity from the left amygdala to bilateral supplementary motor areas, while the MDD-aMCI group showed no significant difference. There was no difference for the pattern of effective connectivity between MDD-aMCI and aMCI groups. (P<0.05, Alphasim corrected; Figure 1, Table 2).

**Table 2:** Significantly altered directional connectivity from and to bilateral amygdala and hippocampus in MDD-aMCI and aMCI patients

	Region	Cluster size	MNI coordinates (mm)			Peak t value
			x	y	z	
**From Left Amygdala**						
aMCI vs Cs	SMA (L/R)	66	-3	9	51	5.91
**From Right Amygdala**						
MDD-aMCI vs Cs	LG/ CG (R)	71	18	-72	-9	5.92
aMCI vs Cs	SMA (L/R)	75	-3	9	51	5.65
MDD-aMCI vs aMCI	LG/ CG (R)	60	9	-81	0	4.41
	SMA (L/R)	86	-6	9	51	-4.50
**To Left Hippocampus**						
MDD-aMCI vs Cs	SPG (L)	40	-18	-72	45	5.42
	SOG/MOG (L)	34	-18	-72	45	5.42
MDD-aMCI vs aMCI	SPG (L)	43	-18	-72	45	5.82
	SOG/MOG (L)	25	-18	-72	45	5.82

Abbreviations: MDD-aMCI, both with Major Depression Disorder and Amnestic Mild Cognitive Impairment; aMCI, Amnestic Mild Cognitive Impairment; Cs, Controls; L, Left; R, Right; LG = Lingual Gyrus; CG, Calcarine Gyrus; SMA, Supplementary Motor Area; SPG, Superior Parietal Gyrus; SOG/MOG, Superior / Middle Occipital Gyrus.

### Effective connectivity to the left and right amygdala

Among the three groups, there was no significant difference for effective connectivity pattern from the whole brain regions to both left and right amygdala (P<0.05, Alphasim corrected).

### Effective connectivity from the left and right hippocampus

There was no difference for the effective connectivity pattern from both left and right hippocampus to other brain regions among the three groups (P<0.05, Alphasim corrected).

### Effective connectivity to the left and right hippocampus

**Figure 2:**
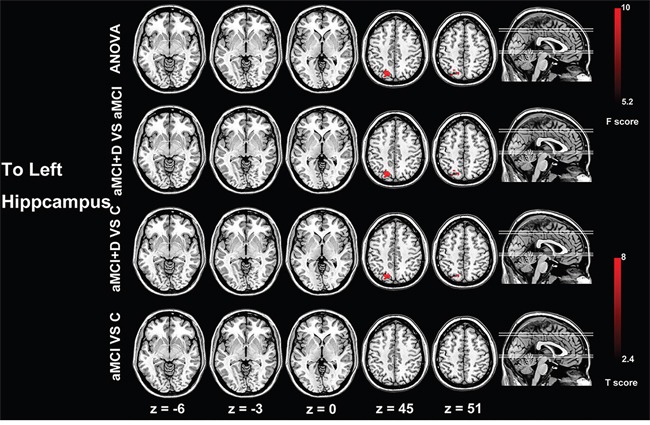
Group differences of effective connectivity based on the seed of left hippocampus ANOVA analysis shows significant difference of effective connectivity from the left superior parietal gyrus and superior and middle occipital gyrus to the left hippocampus. Compared with the aMCI patients and healthy controls, the MDD-aMCI patients have increased effective connectivity from the left superior parietal gyrus and superior and middle occipital gyrus to the left hippocampus. The t value and F value color-coded scale are reported at the right side of the images. Abbreviations: ANOVA, analysis of variance; D, major depressive disorder; aMCI, amnestic mild cognitive impairment; C, healthy control.

The effective connectivity pattern to the left hippocampus was different among the three groups, which were mostly distributed in the left superior parietal gyrus, left superior and middle occipital gyrus, left precuneus and the left inferior parietal lobe. The patients in the MDD-aMCI group showed the increased effective connectivity from the left superior parietal gyrus, superior and middle occipital gyrus, precuneus and the left inferior parietal lobe to the left hippocampus compared with the healthy controls and aMCI groups. The aMCI group showed no different effective connectivity to the left hippocampus compared with the healthy controls. Meanwhile, there was no significant difference for the effective connectivity pattern to the right hippocampus among the three groups (P<0.05, Alphasim corrected) (Figure 2, Table 2).

### Effective connectivity between the seed regions

There was no different effective connectivity between bilateral hippocampus and bilateral amygdala among these three groups. Moreover, the effective connectivity patterns between both ipsilateral and contralateral hippocampus and amygdala were not detected with significant difference among the three groups (P<0.05, Alphasim corrected)

### Correlations results

**Figure 3:**
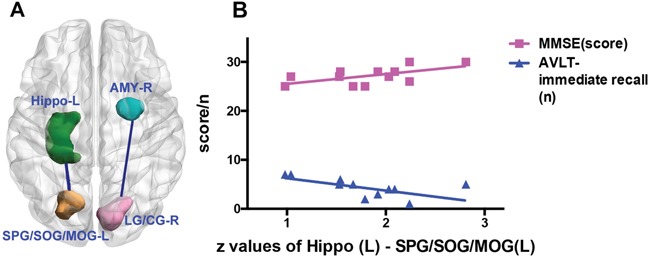
Scatter plots between the extracted z values based on the left hippocampus and neuropsychological scales Brain regions with the green, blue, yellow and pink coded color stand for the left hippocampus, right amygdala, left superior parietal gyrus / superior and middle occipital gyrus (SPG/SOG/MOG) and right lingual and calcarine gyrus (LG/CG), respectively (Figure 3A). Based on the seed of the left hippocampus, the z values of effective connectivity from the left SPG/SOG/MOG to the hippocampus correlate with the scores of MMSE and the numbers of AVLT-immediate recall in the MDD-aMCI group (Figure 3B). Pearson correlation analyses reveals that the z values of the effective connectivity from the left SPG/SOG/MOG to the left hippocampus negatively correlate with the scores of AVLT-immediate recall (r=-0.626, P=0.029), and positively correlate with the scores of MMSE (r= 0.594, P=0.042) (Figure 3B). Abbreviations: L = left; R = right; AMY = amygdala; Hippo = hippocampus; LG/CG = lingual and calcarine gyrus; SPG= superior parietal gyrus; SOG/MOG = superior and middle occipital gyrus; MMSE = Mini-Mental State Examination; AVLT = Auditory Verbal Learning Test.

Pearson correlation analyses revealed that the z values of the effective connectivity from the left superior parietal gyrus and superior and middle occipital gyrus to the left hippocampus negatively correlated with the scores of AVLT-immediate recall (r=-0.626, P=0.029) and positively correlated with the scores of MMSE (r= 0.594; P=0.042) in the patients with MDD-aMCI (Figure 3, Supplementary Table 3). There were no correlations found between the scores of the neuropsychological tests and z values extracted from the following brain regions, i.e., supplementary motor area, superior parietal gyrus, superior and middle occipital gyrus and lingual gyrus, calcarine gyrus, in MDD-aMCI and aMCI groups (Supplementary Table 1 and Table 2).

## DISCUSSION

The present study demonstrated abnormal effective connectivity from the right amygdala and to the left hippocampus in the patients with MDD-aMCI. Specifically, the directional influence of amygdala – occipital-parietal lobe - hippocampus might be the pathway underlying the pathophysiological mechanism of the interaction between MDD and aMCI. Moreover, the increased effective connectivity between the occipital-parietal regions to the left hippocampus was associated with the scores of AVLT in patients with MDD-aMCI.

In this study, MDD-aMCI patients displayed increased effective connectivity from the right amygdala to the right lingual and calcarine gyrus, as well as from the left superior parietal gyrus, superior and middle occipital gyrus to the left hippocampus. The lingual/calcarine gyrus and superior/middle occipital gyrus, as parts of the visual cortex, were involved in visual spatial processing, perception and attention [25–27]. It is worth noting that the alterations of abnormal volume, functional connectivity, metabolism and biochemistry of occipital lobe have been reported in patients with depressive disorder [28–32]. Moreover, the fMRI studies uncovered the distant influences of amygdala on visual cortical activation during emotional face processing, and detected diminished functional connectivity between the amygdala and occipital lobe in patients with MDD-aMCI [31, 32]. Positron emission tomography study has further revealed the reduced serotonin-1A receptor binding potential limited to the amygdala-hippocampus and occipital regions [34]. Lingual gyrus was involved in visual processing, word processing and encoding visual memory. The activation of lingual gyrus was coordinated with memorization when subjects were verbalizing high-emotion words or images [35, 36]. This suggested that lingual gyrus had potential links between amygdala and hippocampus [35–37]. Taken all together, the occipital regions were vulnerable to be suppressed in the patients with MDD-aMCI, which might be related to aberrance in amygdala and hippocampus.

Our study indicated that the occipital regions were influenced by the right amygdala, then had the causal effect on the left hippocampus in patients with MDD and aMCI [38]. First, several anatomical studies in the monkey and diffusion tensor imaging in humans showed direct connections between the occipital and temporal lobe [25, 39], particularly in the structural connection of amygdala – occipital-parietal lobe – hippocampus. Second, the release of acetylcholine in the amygdala of mice positively correlated with performance on the hippocampus-dependent task, which implied that activation of the amygdala would promote processing in other neural systems important for learning and memory [40]. Moreover, greater deficits in the memory domain were found in patients with both MDD and aMCI than aMCI patients [7, 8]. The greater hippocampus dysfunction in MDD-aMCI patients might be interpreted by the causal effects from the amygdala to the hippocampus. Thus, the abnormal effective connectivity of amygdala–occipital lobe–hippocampus loop might be neural basis by which the MDD accelerated the cognitive dysfunction in aMCI patients. The occipital lobe was the relay region by which the amygdala indirectly impaired the hippocampus function, leading to more widespread cognitive impairment in patients with MDD-aMCI. Interestingly, our study found the increased effective connectivity from the bilateral amygdala to bilateral supplementary motor areas in the patients with aMCI compared to the controls. The anatomic-functional links of amygdala-supplementary motor area are closely related to the transformation of emotional experiences into motor actions [40, 41]. Moreover, supplementary motor area is important for midbrain and thalamic circuits implicated in attention [42], motor learning [43] and verbal working memory [44]. The abnormal supplementary motor area was reported in aMCI patients as well [45].

This study has several limitations. Firstly, the small sample size might weaken the statistical power in our study. Further studies with a large cohort are needed in future. Secondly, depression was evaluated by one well-experienced neurologist according to standard method but no quantitative data to classify the severity degree of depression. Thirdly, the use of medication was probably an important confounding factor for the reorganization of brain functional network [46]. Fourthly, it has been reported that the two hemispheres of the human brain differ in their anatomy and function, thus whether there was any asymmetry in effective functional connectivity between the left and right occipital lobe should be taken account into in the future study [47]. Lastly, our study focused on the effect of MDD on aMCI, further studies on the effect of aMCI on MDD are expected in future.

In summary, the present study showed the altered directional effective functional connectivity patterns of the amygdala and hippocampus in MDD-aMCI patients. We postulate that the directional effective functional connectivity of amygdala - occipital-parietal lobe – hippocampus might be the pathway by which MDD inhibits the brain activity in patients with aMCI.

## MATERIALS AND METHODS

### Subjects

All patients and healthy subjects in this study were recruited from the memory clinic of the neurology department of Xuanwu Hospital, Capital Medical University, Beijing, China. A total of 39 subjects (aged 53-83 years) included cognitive normal, non-depressed controls (CN: n=14, 4 males and 10 females, mean age of 64.7 ± 7.0 years), aMCI (n =13, 5 males and 8 females, mean age of 72.7 ± 8.5 years), and aMCI with depression (MDD-aMCI: n =12, 5 males and 7 females, mean age of 69.5 ± 10.3 years). This study was approved by the Medical Research Ethics Committee and Institutional Review Board of Xuanwu Hospital and all participants provided written informed consent. The detailed demographic and clinical data for all the participants are presented in Table 1.

All subjects received detailed clinical and neuropsychiatric assessments characterizing cognitive dysfunction according to previous MDD and aMCI studies [7, 48, 49], including Mini-Mental State Examination (MMSE), Montreal Cognitive Assessment (MoCA) tests, the Auditory Verbal Learning Test (AVLT), Clock Drawing Test (CDT) and Clinical Dementia Rating (CDR) Scale. All the subjects included were right-handedness, scored ≤ 4 on the modified Hachinski ischaemic scale, and MMSE scored ≥ 24 [50]. For healthy controls, CDR=0. The diagnostic assessment of MDD and aMCI were conducted by trained neurologists and neuropsychologists (Y.H with 30 years’ experience in AD) according to the structured Clinical Interview for DSM IV [51] and Petersen’s criteria [52, 53]. Exclusion criteria were as follows: left-handedness, any past and current history of psychiatric disorder, such as psychotic or bipolar disorders; head trauma, drug or alcohol abuse, long-term corticosteroid therapy, MR imaging contraindications; active suicidality; head motions with translation more than 1.0 mm or rotation more than 1.0° during MR examinations. Finally, 14 healthy controls (4 males and 10 females), 13 aMCI patients (5 males and 8 females) and 12 patients with MDD and aMCI (5 males and 7 females) were included in the final analysis.

### MR imaging data acquisition

All subjects were scanned on a 3 Tesla MRI scanner (TIM Trio, Siemens Healthineers, Erlangen, Germany) at Xuanwu Hospital, Capital Medical University. During the MRI scans, all participants were instructed to lie quietly, keep their heads still and eyes closed but be awake. A foam pad was used to minimize head motion. T2 fluid-attenuated inversion-recovery acquisition was conducted to rule out clinically silent lesions, with the parameters as follows: 25 axial slices; slice thickness = 4 mm; slice gap = 1.2 mm; image matrix = 232 × 256; field of view (FOV) = 220 × 220 mm^2^; repetition time [TR] / echo time [TE], 9000 ms/93 ms; flip angle = 130°; inversion time = 2500 ms. rs-fMRI data were acquired using a single-shot, gradient-recalled echo planar imaging sequence (the total time points = 239 sec; TR = 2000 ms, TE = 40 ms, flip angle = 90°, slice number = 28, slice thickness = 4 mm, gap = 1 mm, voxel size = 4×4×4 mm^3^, matrix size = 64×64.

### Data preprocessing

Image preprocessing was conducted using Statistical Parametric Mapping software (SPM8, http://www.fil.ion.ucl.ac.uk/spm/). The first 5 volumes of each set of fMRI data were discarded for the signal equilibrium and subjects’ adaptation to the scanning state. Functional MRI data with the remaining 234 time points were post processed by using the software. The image processing included slice timing, realignment, and head motion correction. Individuals with translation or rotation parameters less than 1.0 mm or 1.0° were included, and there were no significant differences for translation or rotation parameters among the three groups (One-way analysis of variance (ANOVA), both P > 0.05) [40]. The processed data were spatially normalized to the Montreal Neurological Institute space with a voxel size of 3×3×3 mm^3^ by using unified segmentation algorithm, and smoothed by convolution with an isotropic Gaussian kernel of 6 mm. Six head motion parameters and average signals from cerebrospinal fluid, white matter, and whole brain [24], were covariated as well. Lastly, to filter the low-frequency drift and respiratory or cardiac noise, the residual time series were filtered (0.01-0.08 HZ).

### Regions of interest (ROIs) and seed generation

rs-fMRI data were further analyzed using a seed-based functional connectivity algorithm. The bilateral hippocampus and amygdala were selected as the seed regions from Automated Anatomical Labeling provided by the WFU PickAtlas Tool version 3.0 (http://fmri.wfubmc.edu/software/PickAtlas) [54].

### Effective connectivity analysis

The method of granger causality analysis was applied in our study to describe the effective connectivity between the reference time series of the seed regions (left and right amygdala and hippocampus, respectively) and the time series of each voxel within the whole brain. Meanwhile, the study also estimated the granger causal effects between the reference time series within each two seed regions of bilateral amygdala and hippocampus. Our study performed the voxel-wise and ROI-wise granger causality analysis on the residual-based F by using the REST-GCA in the REST toolbox [55]. Granger causality analysis is a technique based on the idea that, given two time series x and y, if knowing the past of y is useful for predicting the future of x then y must have a causal influence on x [56]. In this study, the time series of the bilateral amygdala and hippocampus were defined as the seed time series x, and the time course of voxels within the whole brain are defined as y. The linear direct influence of x on y (*F_x→y_*), and the linear direct influence of y on x (*F_y→x_*) were calculated voxel by voxel across the brain. The residual-based *F* was transformed to normal distributed *F’*, then *F’* of each voxel was standardized to *Z* score (*Z_x→y_* and *Z_y→x_*, subtracting the global mean *F’* values, then being divided by standard deviation) [38].

### Statistical analysis

The demographic or neuropsychiatric data analysis was performed with SPSS version 17.0 (SPSS Inc., Chicago, IL, USA), and SPM8 (statistical parametric mapping, http://www.fil.ion.ucl.ac.uk/spm/) was used to analyze rs-fMRI data. χ2 test was applied to evaluate gender difference among three groups. The Kolmogorov-Smirnov test was conducted to assess the normality of quantitative data. Normally distributed data were expressed as mean ± standard deviation and then assessed by ANOVA or independent sample t tests (the homogeneity of variance of these data was examined by the Bartlett test). Fisher test was employed when the variance was homogeneous, while the Brown-Forsythe approximate variance analysis was applied for the data with heterogeneous variance. Independent samples nonparametric testing was used to analyze the data with non-normal distribution, which were reported as median and inter-quartile range [M (QU-QL)]. If the variance analysis revealed significant differences, post hoc analysis was conducted for inter-group comparisons. P < 0.05 was regarded as a significant difference.

ANOVA testing was performed to detect hippocampus-based functional connectivity differences among the three groups, with age and gender input as covariates. The differences between the groups were then assessed by post hoc analysis, and the results of the analysis were corrected by the AlphaSim program (significance level was set at an uncorrected P < 0.01, with number of clusters > 32, corresponding to a corrected P < 0.05). Subsequently, the z-values of the brain regions with statistical differences at ANOVA testing were extracted and correlated against the scores of the neuropsychological tests. Correlation analyses were performed by using Pearson (normal distribution data) or Spearman correlation analysis (non-normal distribution data). P < 0.05 was regarded as a significant difference.

## SUPPLEMENTARY TABLES



## Abbreviations

AD: Alzheimer’s disease
aMCI: Amnestic MCI
AVLT: Auditory Verbal Learning Test
CDR: Clinical Dementia Rating
CDT: Clock Drawing Test
CN: cognitive normal
GCA: Granger causality analysis
MCI: mild cognitive impairment
MDD: major depression disorder
MMSE: Mini-Mental State Examination
MoCA: Montreal Cognitive Assessment
ROI: Regions of Interest
rs-fMRI: resting-state functional MR imaging
