# Supplementary material for: Altered amygdala and hippocampus effective connectivity in mild cognitive impairment patients with depression: a resting-state functional MR imaging study with granger causality analysis
Source: Oncotarget. 2017 Feb 15;8(15):25021–31. doi: 10.18632/oncotarget.15335 (PMC5421906; doi:10.18632/oncotarget.15335)
Supplement: Supplementary file 1 [file oncotarget-08-25021-s001.pdf]

Altered amygdala and hippocampus effective connectivity in mild cognitive impairment patients with depression: a resting-state functional MR imaging study with granger causality analysis

SUPPLEMENTARY TABLES

Supplementary Table 1: Correlation results between the altered effective connectivity of amygdala (R) - supplementary motor area (bilateral) and neuropsychological performances in aMCI-MDD and aMCI patients

Variables	MDD-aMCI		aMCI	
	r	P	r	P
MMSE/30	-0.120	0.710[e]	0.036	0.908[e]
MOCA/30	-0.098	0.714[d]	-0.058	0.847[d]
AVLT-immediate learning (n)	-0.087	0.789[e]	0. 070	0. 820[e]
AVLT-delayed recall (n)	0.029	0.931[d]	-0.001	0.886[d]
AVLT-recognition (n)	0.093	0.771[d]	0.118	0.698[d]
CDT (score)	-0.150	0.396[d]	0.308	0.305[d]

[d] Spearman rank correlation; [e] Pearson correlation;
MDD-aMCI = Major Depression Disorder and Amnestic Mild Cognitive Impairment; aMCI = Amnestic Mild Cognitive Impairment.
n = number; MMSE = Mini-Mental State Examination; MoCA = Montreal Cognitive Assessment; AVLT = Auditory Verbal Learning Test (AVLT); CDT = Clock Drawing Test.

Supplementary Table 2: Correlation results between the altered effective connectivity of amygdala (R) – lingual and calcarine gyrus (R) and neuropsychological performances in aMCI-MDD and aMCI patients

Variables	MDD-aMCI		aMCI	
	r	P	r	P
MMSE/30	0.147	0.647[e]	-0.190	0.534[e]
MOCA/30	0.046	0.890[d]	-0.220	0.461[d]
AVLT-immediate learning (n)	-0.572	0.052[e]	0.189	0.537[e]
AVLT-delayed recall (n)	0.126	0.694[d]	0.020	0.951[d]
AVLT-recognition (n)	0.345	0.271[d]	0.033	0.916[d]
CDT (score)	-0.094	0.509[d]	0.253	0.246[d]

[d] Spearman rank correlation; [e] Pearson correlation;
MDD-aMCI = Major Depression Disorder and Amnestic Mild Cognitive Impairment; aMCI = Amnestic Mild Cognitive Impairment.
n = number; MMSE = Mini-Mental State Examination; MoCA = Montreal Cognitive Assessment; AVLT = Auditory Verbal Learning Test (AVLT); CDT = Clock Drawing Test.

Supplementary Table 3: Correlation results between the altered effective connectivity of hippocampus (L) – superior parietal gyrus and superior and middle occipital gyrus (L) and neuropsychological performances in aMCI-MDD and aMCI patients

Variables	aMCI-MDD		MCI	
	r	P	r	P
MMSE/30	**0.594**	**0.042**[e]*	-0.354	0.235[e]
MOCA/30	0.077	0.813[d]	-0.006	0.982[d]
AVLT-immediate learning (n)	**-0.626**	**0.029**[e]*	-0.334	0.264[e]
AVLT-delayed recall (n)	0.436	0.157[d]	-0.210	0.423[d]
AVLT-recognition (n)	0.041	0.899[d]	-0.268	0.373[d]
CDT (score)	0.265	0.406[d]	-0.148	0.427[d]

[d] Spearman rank correlation; [e] Pearson correlation;
* $P < 0.05$;
n = number; MMSE = Mini-Mental State Examination; MoCA = Montreal Cognitive Assessment; AVLT = Auditory Verbal Learning Test (AVLT); CDT = Clock Drawing Test.
